# Work–school conflict and turnover intention among Chinese Music University students with part-time jobs: the roles of negative affect and resilience

**DOI:** 10.3389/fpsyg.2026.1849001

**Published:** 2026-06-18

**Authors:** Lingke Zhang, Asheng Aguo, Lijun Huang, XianTing Zhang, Tingting Zhang

**Affiliations:** 1 School of Music and Dance, Xihua University, Chengdu, Sichuan, China; 2 College of Humanities and Management, Guangdong Medical University, Zhanjiang, Guangdong, China; 3 School of Education and Arts, Chongqing College of Humanities, Science and Technology, Hechuan, Chongqing, China

**Keywords:** negative affect, resilience, turnover intention, university students, work-school conflict

## Abstract

**Introduction:**

As increasing numbers of university students engage in part-time work, balancing academic and work demands has become a critical challenge. Work–school conflict (WSC) may be associated with greater negative affect and stronger turnover intention among student workers. This study examined the association between WSC and turnover intention among Chinese music university students with part-time jobs, focusing on the mediating role of negative affect and the moderating role of resilience.

**Methods:**

A cross-sectional survey was conducted with 518 Chinese music university student workers (mean age = 20.27, SD = 1.40, 57.3% females). Validated scales were used to assess WSC, negative affect, turnover intention, and resilience. Structural equation modeling was conducted to test a model in which negative affect was specified as a mediator and resilience as a moderator of the direct and indirect paths.

**Results:**

WSC was positively associated with negative affect (β = 0.36, *p* < 0.001), which in turn was positively associated with turnover intention (β = 0.28, *p* < 0.001), indicating a significant mediating effect of negative affect (β = 0.10, 95% CI [0.04, 0.16]). Resilience moderated only the direct association between WSC and turnover intention (β = −0.13, *p* < 0.001), whereas its moderating effects on the paths from WSC to negative affect and from negative affect to turnover intention were not significant. The positive association between WSC and turnover intention was stronger at low levels of resilience than at high levels of resilience.

**Conclusions:**

These findings suggest that negative affect is one pathway through which WSC is associated with turnover intention and that resilience may weaken the direct association between WSC and turnover intention. Overall, the findings help clarify how role conflict, emotional responses, and personal resources are jointly related to turnover intention among student workers.

## 1 Introduction

Part-time employment has become an increasingly common experience among university students (Jackson, 2024). In addition to providing financial support, part-time work may also contribute to the development of work-related skills and future career opportunities (Douglas and Attewell, 2019). Nevertheless, combining employment with academic study often creates tension between the two roles. One important form of such tension is work-school conflict (WSC), which refers to situations in which work demands interfere with students' academic responsibilities (Almira et al., 2023). Because students generally regard their academic role as primary, the work-school interface is often characterized by a unidirectional pattern in which work undermines school functioning rather than the reverse (Hasyim and Muafi, 2023).

For music university students, the work–school interface may be shaped by demands that are less common in many general university programs. Unlike many general university students, music students must manage not only regular coursework but also intensive artistic training, including regular rehearsals, performances, ensemble activities, examinations, and sustained individual practice (Jääskeläinen et al., 2020). These professional demands often involve heavy time investment, rigid schedules, repeated evaluation, and preparation that cannot easily be postponed or compressed (Okan and Usta, 2021; Jääskeläinen and López-Íñiguez, 2022). As a result, the academic role of music students may be more time-sensitive, performance-based, and demanding than that of many students in less practice-intensive disciplines (Jääskeläinen et al., 2023). Importantly, these demands are not only academic tasks but are also closely tied to professional development and identity formation (Creed et al., 2023). In this context, part-time work may not simply add to students' workload; rather, it may compete directly with highly central training responsibilities and interfere with activities that are difficult to reschedule, such as rehearsals, performances, and individual practice (Park and Sprung, 2015), thereby making WSC particularly salient in this group. Focusing on music university students therefore allows WSC to be examined in a student-employment context where academic demands are highly structured, performance-based, and closely linked to professional development.

One important outcome that may be affected by WSC is turnover intention. In general, turnover intention refers to an individual's conscious willingness or tendency to leave a job and is widely regarded as a proximal predictor of actual turnover behavior (Sun et al., 2025). However, in the context of part-time student employment, its meaning may differ somewhat from that observed among full-time employees. Because student workers usually prioritize their academic role over their work role, the intention to leave a part-time job may reflect not only dissatisfaction with work itself, but also an attempt to reduce role interference and reallocate limited time and energy toward academic demands (Creed et al., 2023; Taylor and Bobadilla Sandoval, 2024). Prior studies have suggested that incompatibility between work and school demands can undermine attitudes toward work, weaken commitment, and increase the desire to quit (Headrick and Park, 2024). Empirical evidence among student workers has likewise linked WSC to stronger turnover intention (Laughman et al., 2016; Noor et al., 2023). This may be especially relevant for student workers because part-time employment is often more peripheral to their primary academic role and may therefore be more readily relinquished when conflicts with academic demands intensify.

Although the association between WSC and turnover intention has been supported in previous studies, the mechanisms underlying this relationship remain insufficiently understood. One possible explanation is that WSC may be associated with stronger turnover intention through its links with negative affect. Negative affect refers to a broad range of unpleasant emotional experiences, such as distress, nervousness, upset, and irritability (Liu et al., 2020). According to Affective Events Theory (AET; Weiss and Beal, 2005), work-related events and experiences can trigger affective reactions, which subsequently shape individuals' work attitudes and behavioral intentions. From an AET perspective, WSC may be understood not simply as a background condition, but as a recurring work-related experience in which job demands repeatedly interfere with academic responsibilities (Wan et al., 2022). For student workers, such interference may occur when work schedules clash with classes, rehearsals, practice, or academic deadlines (Peng et al., 2023). These repeated episodes of role interference may be experienced as affectively salient negative events because they generate frustration, tension, and difficulty in managing competing role demands (Jääskeläinen et al., 2023). As a result, such recurring negative work-related experiences may be associated with negative affect, which in turn may be related to students' intention to leave their part-time jobs.

This argument is also supported by prior empirical findings. Research suggests that students experiencing stronger WSC may be more likely to experience adverse emotional reactions, including negative affect (Peng et al., 2023; Charan et al., 2025). In addition, studies across occupational contexts have found that negative emotional experiences are associated with stronger turnover intention (Laughman et al., 2016; Modaresnezhad et al., 2021; Qin et al., 2023). Although few studies have directly examined negative affect as a mediator between WSC and turnover intention, previous findings offer indirect support for this pathway. For example, previous studies found that burnout significantly mediated the association between WSC and turnover intention (Laughman et al., 2016; Noor et al., 2023). In addition, another study showed that WSC was positively associated with turnover intention through work stress (Hasyim and Muafi, 2023). Although these studies did not directly examine negative affect, they provide indirect support for the possibility that adverse emotional reactions may help explain why WSC is related to turnover intention. However, these previous explanations have largely framed the WSC–turnover intention association in terms of broader strain accumulation. By contrast, examining negative affect allows the present study to conceptualize this association as a more proximal affective process, thereby providing a closer test of the event–affect–outcome logic proposed by AET. This approach reframes the WSC–turnover intention link as an affective process, rather than treating it only as a consequence of broad strain accumulation. Based on this theoretical and empirical reasoning, negative affect may be one pathway through which WSC is associated with turnover intention. Accordingly, we proposed the following hypothesis:

H1: Negative affect would mediate the association between WSC and turnover intention.

Resilience refers to an individual's capacity to adapt positively and recover effectively when facing adversity, stress, or challenging circumstances (Connor and Davidson, 2003). It helps individuals cope with pressure across different life domains, regulate distress, and maintain psychological functioning under demanding conditions (Hou et al., 2021; Lu et al., 2024a). In the present study, resilience may moderate the associations among WSC, negative affect, and turnover intention. According to Conservation of Resources (COR) theory, individuals strive to obtain, retain, and protect valued resources, and strain is likely to arise when these resources are threatened or depleted (Demerouti, 2025). In the context of student employment, persistent WSC may consume time, energy, attention, and self-regulatory capacity, thereby making continued employment increasingly difficult to sustain (Park and Sprung, 2015; Peng et al., 2023). Students with higher resilience, however, may be better able to preserve functioning, recover from role-related stress, and cope with ongoing interference between work and school (Waugh and Sali, 2023; Egan et al., 2024). This possibility is broadly consistent with prior research showing that resilience can moderate the extent to which work-related conflict, workload, stress, or professional quality of life are associated with turnover-related outcomes (Lanz and Bruk-Lee, 2017; Mousavi mahdi et al., 2023; Al Yahyaei et al., 2025), although findings have not always been fully consistent across occupational groups. From this perspective, resilience may weaken the extent to which persistent WSC is associated with turnover intention. More importantly, the role of resilience in the present study is not conceptualized simply as that of a generic buffering factor. Rather, drawing on COR theory, we argue that resilience may function as a personal resource that shapes whether persistent WSC is associated with stronger turnover intention. Examining resilience at this stage helps clarify when WSC is more strongly or weakly associated with withdrawal intention. Thus, we proposed the following hypothesis:

H2: Resilience would moderate the direct association between WSC and turnover intention, such that this positive association is weaker at higher levels of resilience.

Resilience may also condition the emotional pathway linking WSC to turnover intention. Beyond its potential role in the direct WSC–turnover intention association, resilience may also shape whether WSC is associated with stronger negative affect and whether such affective responses are further linked to turnover intention. On the one hand, WSC may increase negative affect because interference between work and school responsibilities can generate frustration, tension, and emotional strain (Park and Sprung, 2015; Peng et al., 2023). Yet students with higher resilience may be better able to regulate stress reactions and maintain emotional stability under pressure, suggesting that the positive association between WSC and negative affect may be weaker at higher levels of resilience (Afek et al., 2021; Egan et al., 2024). Prior research has likewise shown that resilient coping can weaken the associations between inter-role conflict and adverse emotional outcomes such as stress, anxiety, and depression (de Sousa et al., 2024), suggesting that resilience may similarly reduce the extent to which WSC is associated with negative affect. Accordingly, we proposed the following hypothesis:

H3: Resilience would moderate the association between WSC and negative affect, such that this positive association is weaker at higher levels of resilience.

Negative affect may also be associated with stronger turnover intention because emotional distress can make continued work involvement feel more burdensome and less sustainable (Jasiński and Derbis, 2022; Qin et al., 2023). At the same time, resilience has been associated more broadly with better emotional adjustment, affect regulation, and recovery from adversity, which is consistent with the possibility that students with higher resilience may be less likely to allow emotional distress to develop into withdrawal intention (Al Yahyaei et al., 2025). Thus, resilience may also weaken the positive association between negative affect and turnover intention. Overall, although existing studies have mainly focused on conventional employee samples rather than student workers, and have rarely examined whether resilience moderates the associations among WSC, negative affect, and turnover intention within the same framework, the available evidence provides a reasonable basis for testing these multiple moderating roles among student workers. Accordingly, we proposed the following hypothesis:

H4: Resilience would moderate the association between negative affect and turnover intention, such that this positive association is weaker at higher levels of resilience.

**Figure 1:**
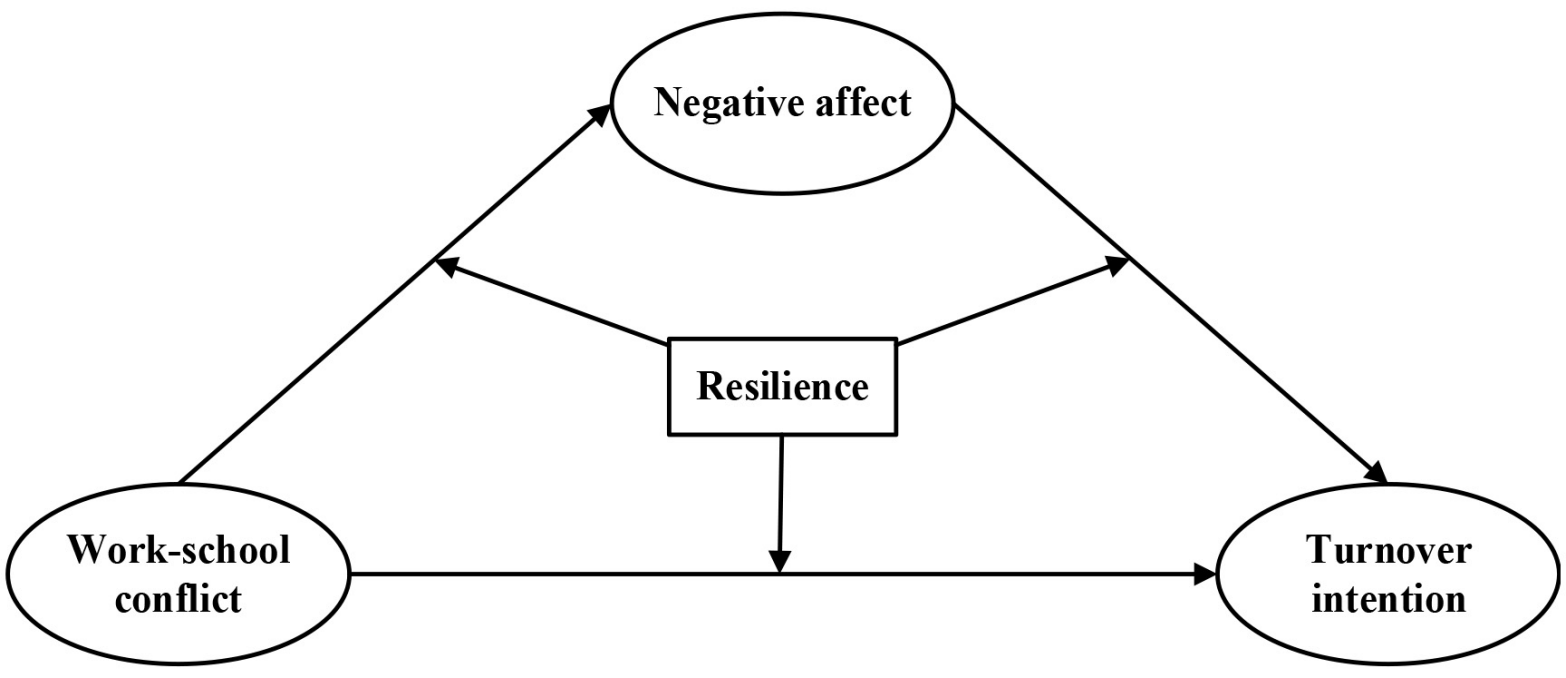
The hypothesized moderated mediation model.

Drawing on the literature reviewed above, the present study examined a moderated mediation framework linking WSC, negative affect, resilience, and turnover intention among Chinese music university students with part-time jobs. Specifically, we proposed that negative affect would mediate the association between WSC and turnover intention, and that resilience would moderate the direct and indirect links among WSC, negative affect, and turnover intention. In doing so, the present study aimed to clarify the WSC–turnover intention association as a more proximal affective process and to examine whether a personal resource operates across different stages of that process. The hypothesized moderated mediation model is presented in Figure 1.

## 2 Methods

### 2.1 Participants and data collection

This study employed a cross-sectional survey design. Data were collected in September 2025 from undergraduate music students at four universities in Sichuan, China. Eligible participants were students who were currently engaged in part-time work during the academic semester. The survey was administered online through Wenjuanxing, a widely used survey platform in China. With the assistance of faculty members and administrative staff, the survey link was distributed to eligible students through university communication channels, including class-based online groups. Before beginning the survey, participants were provided with a brief introduction to the study, including its purpose, procedures, confidentiality protections, and the voluntary nature of participation. They were also informed that their responses would be used for research purposes only and would be analyzed anonymously. Electronic informed consent was obtained from all participants before they accessed the questionnaire.

To improve data quality, each participant was allowed to submit the questionnaire only once. In addition, all items were set as mandatory on the online survey platform, so no item-level missing data were generated. A total of 544 undergraduate music students with part-time work experience completed the survey. After data collection, the responses were screened for validity as part of the data cleaning process. Cases showing logical inconsistencies, obviously careless response patterns, or other invalid response features were excluded from the final dataset. After excluding 26 invalid responses, a final sample of 518 participants was retained for analysis. The study protocol was approved by the research ethics committee of Xihua University (Approval No. XH20250124-01).

### 2.2 Measures

#### 2.2.1 Demographic variables

Demographic variables were collected, including age, sex, academic year, working hours per week, and perceived family financial situation.

#### 2.2.2 Turnover intention

Turnover intention was assessed using the three-item turnover intention scale (Sjöberg and Sverke, 2000). This scale captures a general intention to leave one's current job and has been applied in prior studies involving part-time working student populations (Noor et al., 2023), supporting its applicability in the present context. A sample item is “I feel that I could leave this job”. Participants rated each item on a five-point Likert scale ranging from 1 (strongly disagree) to 5 (strongly agree). The three items were summed to create a total score ranging from 3 to 15, with higher scores indicating stronger turnover intention. The Cronbach's α of the subscale was 0.88 in this study.

#### 2.2.3 Negative affect

Negative affect was assessed using the negative affect subscale of the International Positive and Negative Affect Schedule Short Form (I-PANAS-SF) (Thompson, 2007). The negative affect subscale consists of five items: upset, hostile, ashamed, nervous, and afraid. Participants were asked to indicate the extent to which they had experienced each emotion during the past week on a five-point Likert scale (1 = very slightly or not at all to 5 = extremely). The five items were summed to obtain a total score ranging from 5 to 25, with higher scores indicating higher levels of negative affect. The scale has been validated among Chinese populations and shown acceptable psychometric properties (Liu et al., 2020). The Cronbach's α of the subscale was 0.88 in this study.

#### 2.2.4 WSC

WSC was assessed using the five-item Work-School Conflict Scale (Markel and Frone, 1998). A sample item is,

“My job demands and responsibilities interfere with my school work.”

It was rated on a five-point Likert scale ranging from 1 (never) to 5 (very frequently). The five items were summed to create a total score ranging from 5 to 25, with higher scores indicating more severe WSC. It has been validated in Chinese university students and shown acceptable psychometric properties (Wei, 2018). In the present study, the Cronbach's α of the scale was 0.88.

#### 2.2.5 Resilience

Resilience was assessed using the two-item abbreviated version of the Connor-Davidson Resilience Scale (CD-RISC-2; Vaishnavi et al., 2007). This brief measure has been validated in Chinese populations and demonstrated acceptable psychometric properties (Ni et al., 2016). It has also been widely applied in prior studies involving Chinese samples across different contexts (Lai et al., 2020; Hou et al., 2021; Zhang et al., 2021; Lu et al., 2024b; Liu et al., 2026; Sun et al., 2026), including research on Chinese music university students (Sun et al., 2026), supporting its applicability in contexts similar to the present study. In the present study, resilience was conceptualized as a general personal resource within a COR framework rather than as a multidimensional construct. Accordingly, a brief measure was considered appropriate for capturing overall resilience in the structural model. The two items were “able to adapt to change” and “tend to bounce back after illness or hardship”. It was rated on a five-point Likert scale (0 = not true at all to 4 = true nearly all the time). The two items were summed to produce a total score ranging from 0 to 8, with higher scores indicating higher levels of resilience. The Cronbach's α of the scale was 0.86 in this study.

### 2.3 Data analysis

Descriptive statistics were first computed for participants' characteristics. Specifically, frequencies and percentages were reported for categorical variables, whereas means and standard deviations were reported for continuous variables. The distributional properties of the study variables were also examined by inspecting skewness and kurtosis values to assess normality. In addition, tolerance values and variance inflation factors (VIFs) were calculated to assess multicollinearity. Because all focal variables were measured using self-report questionnaires, common method bias was assessed in three ways: First, Harman's single-factor test was conducted to examine whether a single factor accounted for most of the covariance among the measures. Second, a series of alternative measurement models, including single-factor, two-factor, three-factor, and four-factor models, were compared. Third, a latent common method factor test was conducted by adding a common latent factor to the measurement model to examine whether model fit changed substantially after accounting for potential method variance. Pearson correlation coefficients were then computed for the main variables.

To test the hypothesized model, structural equation modeling (SEM) was conducted. Specifically, WSC, negative affect, and turnover intention were modeled as latent variables using their respective scale items as indicators, whereas resilience was treated as an observed variable in the moderation analyses. First, the adequacy of the measurement model was evaluated by examining the overall model fit and the standardized factor loadings of the latent variables. The structural model was then estimated to test the mediating role of negative affect in the association between WSC and turnover intention, as well as the moderating role of resilience in the paths linking WSC, negative affect, and turnover intention. Indirect effects were evaluated using bias-corrected 95% confidence intervals (CIs) based on 5,000 bootstrap samples, and an indirect effect was considered significant if the CI did not include zero. All continuous variables involved in the interaction terms were mean-centered before analysis to reduce multicollinearity and facilitate interpretation. Sex, age, academic year, working hours per week, and perceived family financial situation were included as control variables. Model fit was assessed using multiple fit indices, including the chi-square to degrees of freedom ratio (χ^2^/df), the comparative fit index (CFI), the Tucker–Lewis index (TLI), the root mean square error of approximation (RMSEA), and the standardized root mean square residual (SRMR). A model was considered to have acceptable fit when χ^2^/df was below three, CFI and TLI were above 0.90, and RMSEA and SRMR were below 0.08 (Kline, 2016). Standardized coefficients (β) are reported in the Results.

Statistical significance was set at *p* < 0.05 (two-tailed). Descriptive statistics, correlations, skewness and kurtosis values, and multicollinearity diagnostics were computed using IBM SPSS Statistics for Windows, version 26.0 (IBM Corp., Armonk, NY, USA), and SEM analyses were conducted in Mplus 8.3.

## 3 Results

### 3.1 Participants' characteristics

A total of 518 undergraduate music students with part-time work experience were included in the final analysis. As shown in Table 1, 221 (42.7%) were male and 297 (57.3%) were female. Regarding academic year, 125 (24.1%) were freshmen, 141 (27.2%) were sophomores, 166 (32.0%) were juniors, and 86 (16.6%) were seniors. In terms of perceived family financial situation, 154 (29.7%) participants reported poor or very poor financial status, 267 (51.5%) reported average financial status, and 97 (18.7%) reported good or very good financial status. The mean age of the participants was 20.27 years (SD = 1.40), and the average working hours per week was 14.16 (SD = 5.58).

**Table 1:** Participants' characteristics.

Categorical variable	*n*	%
Sex		
Male	221	42.7
Female	297	57.3
Academic year		
Freshmen	125	24.1
Sophomores	141	27.2
Juniors	166	32.0
Seniors	86	16.6
Perceived family financial situation		
Very poor/poor	154	29.7
Average	267	51.5
Good/very good	97	18.7
Continuous variable	Mean	SD
Age	20.27	1.40
Working hours per week	14.16	5.58

The means (SD) of the main study variables are presented in Table 2. The mean scores of WSC, negative affect, turnover intention, and resilience were 11.61 (SD = 3.74), 8.59 (SD = 2.35), 6.36 (SD = 1.98), and 4.07 (SD = 2.08), respectively.

**Table 2:** Mean, SD, and Pearson correlations.

Variable	1	2	3	4
1. Work-school conflict	1			
2. Negative affect	0.35^***^	1		
3. Turnover intention	0.43^***^	0.45^***^	1	
4. Resilience	−0.15^***^	−0.32^***^	−0.23^***^	1
Mean	11.61	8.59	6.36	4.07
SD	3.74	2.35	1.98	2.08
Range	5–25	5-25	3–15	0–8

^***^p < 0.001.

### 3.2 Normality and multicollinearity diagnostics

To examine whether the data met the assumptions for the subsequent analyses, we first inspected the distributional properties of the study variables. As shown in Supplementary Table 1, the skewness values ranged from −0.36 to 0.43 and the kurtosis values ranged from −0.92 to 0.61, indicating no serious departures from normality. In addition, multicollinearity diagnostics showed that tolerance values ranged from 0.46 to 0.67 and variance inflation factor (VIF) values ranged from 1.49 to 2.18 (see Supplementary Table 1). These results suggest that multicollinearity was not a serious concern in the present study.

### 3.3 Common method bias test

Because all focal variables were measured using self-report questionnaires, common method bias was examined by comparing a series of alternative measurement models. First, Harman's single-factor test showed that the first unrotated factor accounted for 25.32% of the total variance, which was below the commonly used 40% threshold. Second, as shown in Table 3, the hypothesized four-factor model provided a substantially better fit to the data (χ^2^ = 239.05, df = 84, χ^2^/df = 2.85, CFI = 0.957, TLI = 0.933, RMSEA = 0.047, SRMR = 0.035) than the single-factor model (χ^2^ = 988.29, df = 90, χ^2^/df = 10.98, CFI = 0.654, TLI = 0.617, RMSEA = 0.134, SRMR = 0.076), as well as the two-factor and three-factor models. Third, a latent common method factor test was conducted by adding a common latent factor to the measurement model. The original four-factor measurement model showed good fit (CFI = 0.960, TLI = 0.950, RMSEA = 0.045, SRMR = 0.031). After inclusion of the common latent factor, model fit improved only slightly (CFI = 0.965, TLI = 0.957, RMSEA = 0.039, SRMR = 0.021), and the substantive factor loadings remained essentially unchanged. These results suggest that common method bias was unlikely to have substantially affected the measurement model or fully account for the observed associations.

**Table 3:** Comparison of alternative measurement models for assessing common method bias.

Model	χ^2^	df	χ^2^/df	CFI	TLI	RMSEA	SRMR
Single-factor model	988.29	90	10.98	0.654	0.617	0.134	0.076
Two-factor model	740.78	89	8.32	0.759	0.703	0.108	0.069
Three-factor model	593.89	87	6.82	0.817	0.763	0.076	0.042
Four-factor model	239.05	84	2.85	0.957	0.933	0.047	0.035

Single-factor model: all items loaded onto one latent factor.

Two-factor model: turnover intention items loaded onto one factor, and work-school conflict, negative affect, and resilience items loaded onto a second factor.

Three-factor model: turnover intention items loaded onto one factor, work-school conflict items loaded onto a second factor, and negative affect and resilience items loaded onto a third factor.

Four-factor model: turnover intention, work-school conflict, negative affect, and resilience were specified as four separate latent factors.

CFI, comparative fit index; TLI, Tucker-Lewis index; RMSEA, root mean square error of approximation; SRMR, standardized root mean square residual.

### 3.4 Correlations

WSC was positively correlated with negative affect (*r* = 0.35, *p* < 0.001) and turnover intention (*r* = 0.43, *p* < 0.001). Negative affect was also positively correlated with turnover intention (*r* = 0.45, *p* < 0.001). In contrast, resilience was negatively correlated with work-school conflict (*r* = −0.15, *p* < 0.001), negative affect (*r* = −0.32, *p* < 0.001), and turnover intention (*r* = −0.23, *p* < 0.001). This information is presented in Table 2.

### 3.5 Mediation and moderation results

**Figure 2:**
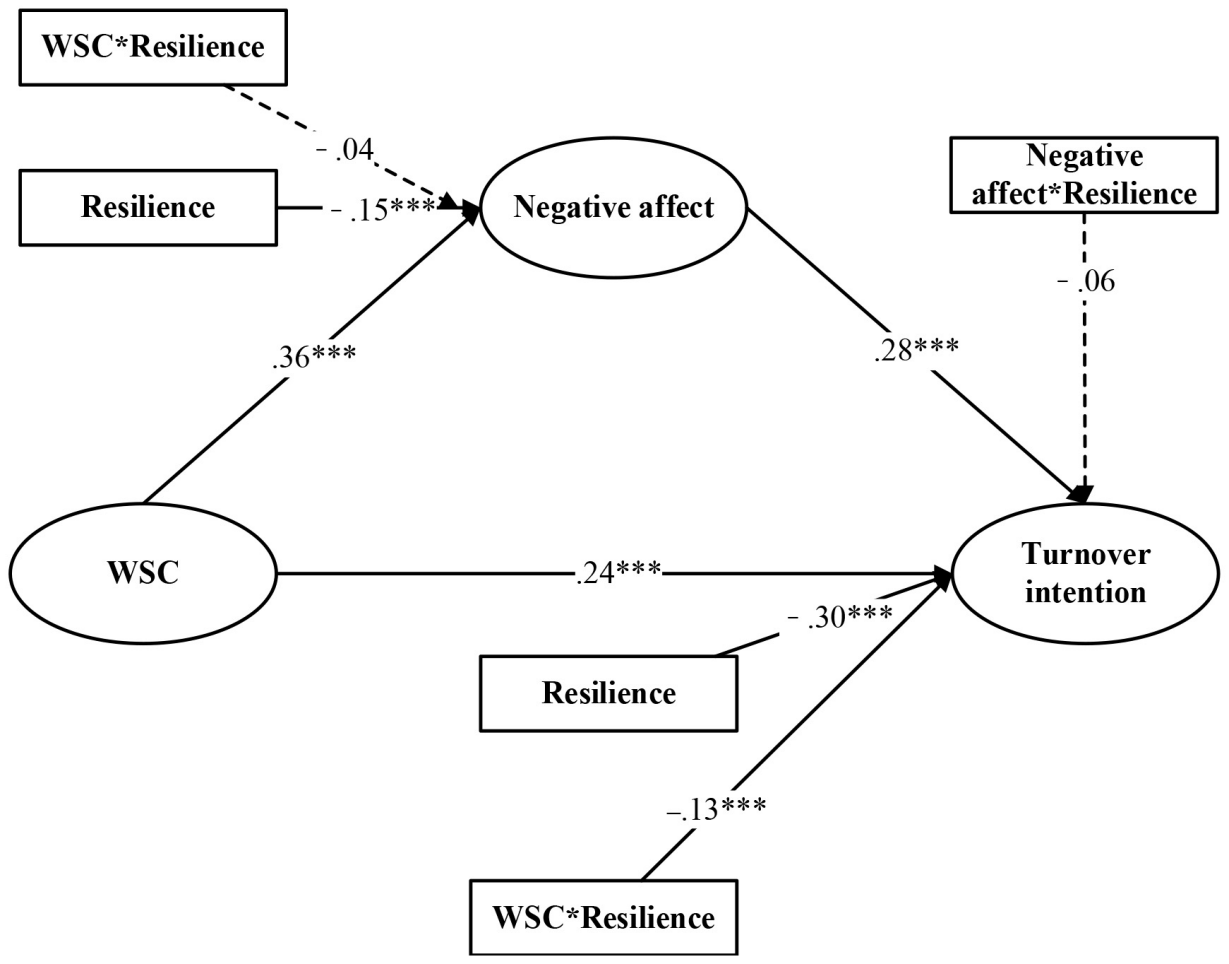
Structural equation model of the associations among WSC, negative affect, resilience, and turnover intention. WSC, work-school conflict; Standardized path coefficients are presented. Solid lines indicate significant paths, whereas dashed lines indicate non-significant paths. ***indicates statistical significance at *p* < 0.001.

Structural equation modeling was conducted to test the hypothesized model. The standardized factor loadings were all significant and ranged from 0.79 to 0.87 for WSC, 0.76–0.91 for negative affect, and 0.81–0.88 for turnover intention, supporting the adequacy of the measurement model. The overall model fit the data well (χ^2^/df = 2.52, CFI = 0.964, TLI = 0.951, RMSEA = 0.034, SRMR = 0.028). As shown in Table 4 and Figure 2, WSC was positively associated with negative affect (β = 0.36, SE = 0.04, *p* < 0.001), and negative affect was positively associated with turnover intention (β = 0.28, SE = 0.04, *p* < 0.001). WSC also had a significant positive association with turnover intention (β = 0.24, SE = 0.05, *p* < 0.001). Bootstrapping further showed that the indirect effect of WSC on turnover intention through negative affect was significant (β = 0.10, SE = 0.03, 95% CI [0.04, 0.16]), supporting the mediating role of negative affect.

**Table 4:** Structural equation modeling results for the mediation and moderated mediation analyses.

Predictor	Negative affect		Turnover intention	
	β (SE)	*p*	β (SE)	*p*
Covariate				
Sex	−0.05 (0.07)	0.432	0.04 (0.06)	0.432
College year	0.02 (0.04)	0.625	−0.02 (0.03)	0.448
Perceived family financial situation	−0.11 (0.05)	0.019	−0.02 (0.01)	0.095
Age	0.01 (0.02)	0.719	−0.02 (0.02)	0.348
Working hours per week	−0.01 (0.01)	0.518	−0.01 (0.01)	0.758
Main variable				
Work-school conflict	0.36 (0.04)	< 0.001	0.24 (0.05)	< 0.001
Negative affect			0.28 (0.04)	< 0.001
Resilience	−0.15 (0.04)	< 0.001	−0.30 (0.04)	< 0.001
Interaction				
Work-school conflict^*^Resilience	−0.04 (0.03)	0.226	−0.13 (0.03)	< 0.001
Negative affect^*^Resilience			−0.06 (0.04)	0.101
*R* ^2^	0.39		0.53	

Standardized coefficients are presented. Work-school conflict, negative affect, and turnover intention were modeled as latent variables, whereas resilience was treated as an observed variable. All continuous variables involved in the interaction terms were mean-centered prior to analysis. *R*^2^ values indicate the proportion of variance explained in the endogenous variables.

^*^indicates an interaction term.

Resilience was negatively associated with both negative affect (β = −0.15, SE = 0.04, *p* < 0.001) and turnover intention (β = −0.30, SE = 0.04, *p* < 0.001). Of the three interaction terms tested, only the interaction between WSC and resilience significantly predicted turnover intention (β = −0.13, SE = 0.03, *p* < 0.001). The interactions between WSC and resilience in predicting negative affect (β = −0.04, SE = 0.03, *p* = 0.226) and between negative affect and resilience in predicting turnover intention (β = −0.06, SE = 0.04, *p* = 0.101) were not significant. These findings indicate that resilience moderated only the direct association between WSC and turnover intention, but not the two paths involving negative affect.

**Figure 3:**
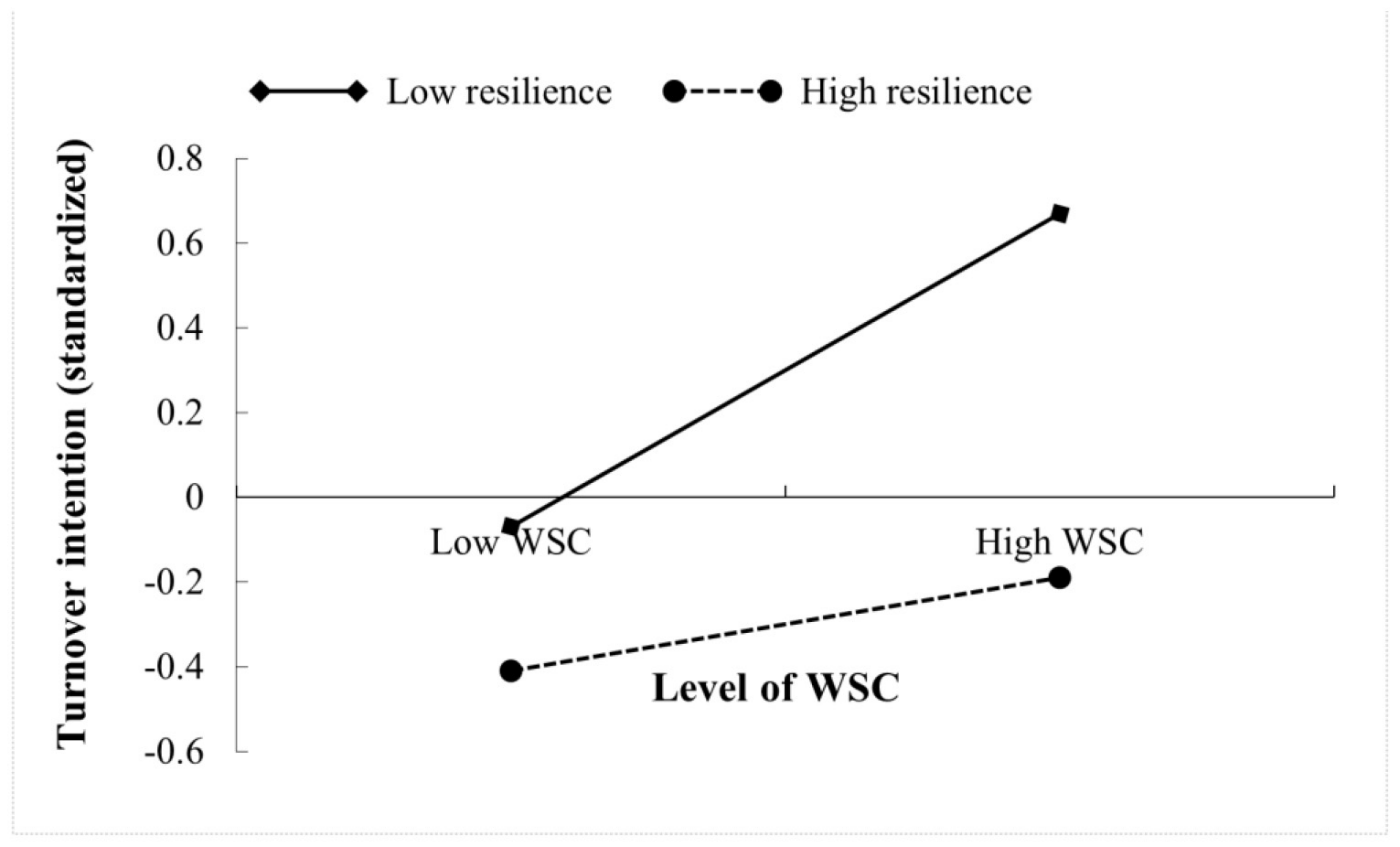
Simple slope plot of the moderating effect of resilience on the association between work–school conflict and turnover intention. Low and high resilience represent values at 1 SD below and above the mean, respectively. The plot is based on standardized values. WSC, work-school conflict.

Simple slope analysis further illustrated the significant interaction between WSC and resilience in predicting turnover intention (Figure 3). Specifically, the slope of WSC on turnover intention was stronger at low levels of resilience (−1 SD; simple slope β = 0.37) than at high levels of resilience (+1 SD; simple slope β = 0.11). This pattern indicates that the positive association between WSC and turnover intention became weaker as resilience increased.

## 4 Discussion

This study examined how WSC was associated with turnover intention among Chinese part-time music university students, focusing on the mediating role of negative affect and the moderating role of resilience. The results indicated that negative affect significantly mediated the association between WSC and turnover intention. In addition, resilience weakened the direct association between WSC and turnover intention. However, resilience did not significantly moderate the association between WSC and negative affect or the association between negative affect and turnover intention. These findings suggest that the association between WSC and turnover intention is linked not only to competing role demands themselves, but also to how those demands are experienced emotionally and how they are shaped by personal resources. More importantly, the present study clarifies the WSC–turnover intention association as a more proximal affective process and suggests that resilience may play a more selective role than originally expected. By situating this process among music university students with part-time jobs, the study also extends WSC research to a structured, performance-based student-employment context in which academic demands are closely tied to professional development.

Our results support H1 and confirm that negative affect significantly mediates the relationship between WSC and turnover intention. This finding is consistent with AET (Weiss and Beal, 2005), which proposes that affective reactions to work-related events shape subsequent attitudes and behavioral outcomes. In the present study, WSC may be understood, from an AET perspective, as a recurring work-related experience in which job demands repeatedly disrupt students' academic responsibilities (Weiss and Beal, 2005). Although WSC is not a single discrete event, its repeated occurrence in everyday student employment may function as a series of affectively meaningful negative episodes that elicit unpleasant emotional states (Peng et al., 2023). These emotional reactions, in turn, may be associated with a stronger likelihood that students will consider leaving their part-time jobs. Previous research has often conceptualized inter-role conflict as a precursor to broader forms of psychological strain, such as burnout or work stress (Laughman et al., 2016; Noor et al., 2023). While these studies have yielded valuable insights, they tend to emphasize relatively diffuse or cumulative forms of distress. By identifying negative affect as a mediator, the present study adds greater conceptual precision to the emotional pathway linking WSC to turnover intention. Compared with burnout or chronic work stress, negative affect may capture a more immediate response to role conflict (Maslach and Leiter, 2016; Edú-Valsania et al., 2022). The present findings therefore help move the explanation of the WSC–turnover intention link from a broad strain-based account toward a more proximal affective process account. This interpretation suggests that student workers may begin to think about leaving not only after strain has accumulated into more chronic forms such as burnout, but also at an earlier stage, when repeated work-related interference is already shaping their emotional experience.

What seems to matter here is not only that WSC is stressful, but that it may alter how students feel about the sustainability of continuing to work. The present findings therefore help refine the application of AET to work-school research by showing that emotional responses to role conflict may be one reason why student workers begin to disengage psychologically from their jobs (Jasiński and Derbis, 2022). In student employment, conflict may not always take the form of severe burnout, but it may still be associated with repeated negative emotional experiences and stronger turnover intention. This point may be especially relevant in student employment, where work is only one part of a broader role structure and where decisions about staying or leaving a job are often made under ongoing academic pressure. Accordingly, the study contributes by clarifying the WSC–turnover intention association through a mechanism that is more closely aligned with the event–affect–outcome logic of AET.

Previous applications of AET have largely focused on conventional organizational settings, examining how workplace events influence outcomes such as job satisfaction, organizational citizenship behavior, or employee withdrawal (Laughman et al., 2016; Noor et al., 2023). The present findings extend this line of work by showing that affective processes may also operate in student-employment contexts, where work is only one part of a broader role structure. For student workers, especially those in music programs, the consequences of work-related events may be intensified by the fact that academic obligations are themselves highly structured and demanding (Miksza et al., 2021). Practice, rehearsal, performance preparation, and evaluation may already place substantial emotional demands on students, so when work interferes with school responsibilities, the emotional consequences of this interference may be particularly salient (Okan and Usta, 2021; Sokoli et al., 2022). We did not directly compare music students with other student groups, so this point should be interpreted cautiously. Nevertheless, the present findings suggest that negative affect may be particularly relevant in structured training contexts where academic demands are intensive, time-sensitive, and closely tied to professional development.

Our moderation analysis supported H2 by showing that resilience significantly moderated the direct link between WSC and turnover intention, such that the positive relationship was weaker among student workers who reported higher levels of resilience. This suggests that the consequences of WSC are not uniform across students. When students experience WSC, they may need to invest additional time, energy, and self-regulatory effort to manage the demands of both roles (Wan et al., 2022; Creed et al., 2023). Over time, this may increase strain and make withdrawal from work appear to be a practical way to reduce pressure (Shah et al., 2022). However, students with higher resilience may be better able to regulate their emotions, recover from setbacks, and maintain functioning despite temporary role interference (Troy et al., 2023). As a result, WSC may be less strongly associated with turnover intention among students with higher resilience.

At the same time, the consequences of WSC appear to depend partly on students' internal coping resources (Wan et al., 2022). In this respect, resilience may serve as a personal resource that helps students absorb the emotional and functional disruption associated with WSC without immediately interpreting the situation as unmanageable (Fang et al., 2024). By contrast, students with lower resilience may have fewer internal resources available to cope with such conflict and may therefore be more likely to respond to WSC with stronger turnover intention (Yi et al., 2024). The significant interaction found in the present study supports this interpretation, and further suggests that resilience plays a buffering role not by eliminating conflict itself, but by weakening its direct behavioral consequences. Theoretically, this finding extends the role of resilience beyond a generic positive individual difference and supports its interpretation as a boundary condition within a COR framework. More specifically, the result suggests that personal resources may shape whether conflict is associated with withdrawal intention, rather than simply reducing all adverse consequences of conflict in a uniform way.

By contrast, H3 and H4 were not supported. Resilience did not significantly moderate the association between WSC and negative affect, nor did it significantly moderate the association between negative affect and turnover intention. Thus, the moderated mediation pattern was not supported. One possible interpretation is that resilience may be more relevant to how students respond behaviorally to role conflict than to whether they experience negative affect in the first place (Al Yahyaei et al., 2025). In other words, higher resilience may not necessarily prevent student workers from feeling distressed when work interferes with school responsibilities, but the association between such conflict and turnover intention may be weaker among those with higher resilience (Poku et al., 2025). Similarly, once negative affect is present, its association with turnover intention may reflect a broader emotional burden that is not easily buffered by resilience alone (Emerson et al., 2023). These non-significant findings suggest that the protective role of resilience in the present study was more specific than initially hypothesized, and appeared to operate mainly on the direct association between WSC and turnover intention rather than across the full emotional pathway. This pattern is theoretically informative because it indicates that the role of resilience may be stage-specific rather than globally protective. In other words, personal resources may not buffer every part of the WSC process to the same extent. Instead, resilience may be especially relevant at the stage where students interpret and respond to conflict in terms of whether to remain in or withdraw from work (Al Yahyaei et al., 2025). This pattern refines the role of resilience within a COR framework, suggesting that its protective function may be more selective than uniformly generalized across all paths.

Much of the prior literature has emphasized external conditions, such as organizational support, schedule flexibility, or supervisor behavior, when explaining why workers stay or leave (Moen et al., 2017; Scanlan and Still, 2019). These factors remain important, but the present findings suggest that internal psychological resources also deserve closer attention (Cabrera-Aguilar et al., 2023). This does not mean that turnover intention should be individualized or reduced to a matter of personal strength (Bryant and Aggleton, 2025). Rather, it indicates that the consequences of WSC are jointly shaped by situational demands and personal capacities for adaptation (Wan et al., 2022). This is particularly relevant for student workers, who often operate with relatively limited autonomy and under considerable role pressure (Creed et al., 2023). Resilience therefore complements, rather than replaces, structural explanations of turnover intention. Taken together, the present findings suggest that the WSC process is best understood neither as a purely situational problem nor as a purely individual one, but as a process in which role conflict, affective responses, and personal resources are jointly associated with withdrawal intention.

The focus on music university students also matters for how the findings are interpreted. These students face a structured student-employment context characterized by intensive training demands, rigid schedules, repeated evaluation, and strong performance pressures (Jääskeläinen et al., 2023). Work-related interference may therefore disrupt not only general academic tasks but also identity-relevant and professionally important activities, such as rehearsals, performances, and sustained individual practice (Okan and Usta, 2021). This contextual focus extends existing WSC research by showing how WSC is associated with affective responses and turnover intention in a setting where academic demands are highly structured, time-sensitive, and central to professional development (Jääskeläinen and López-Íñiguez, 2022). It also offers a useful setting for examining the event–affect–outcome logic of AET and the resource-based processes described by COR theory.

It is also worth noting that the buffering role of resilience should not be overstated. Although students with higher resilience were less likely to show a strong positive association between WSC and turnover intention, resilience should not be viewed as a substitute for reducing role conflict itself (Rees et al., 2015). A focus on resilience alone may risk shifting responsibility away from work and educational environments and onto students as individuals (Rees et al., 2015). The present findings are better interpreted as showing that resilience can mitigate the consequences of WSC, not that it resolves the underlying conflict (Cabrera-Aguilar et al., 2023). Practically, this means that interventions should not only aim to strengthen students' coping resources, but also to reduce avoidable sources of conflict where possible (Van Heerden et al., 2022). Supporting student workers effectively will likely require attention to both sides of the equation: the demands they face and the resources they can draw upon in handling those demands.

Our findings yield several practical implications for both employers and universities. Because negative affect was a significant mediator, reducing turnover intention may require attention to students' emotional experiences, not only to scheduling or workload issues. Universities may therefore consider offering support programs specifically designed for students who combine study and employment, such as emotion regulation training, stress management workshops, or accessible counseling services (Waterhouse and Samra, 2026). The moderating role of resilience also suggests that strengthening adaptive coping resources may be helpful. Interventions such as peer support, cognitive reappraisal training, and resilience-building programs may help reduce the extent to which WSC is associated with turnover intention (Ansari and Iqbal, 2025). Employers, meanwhile, may need to recognize that student workers often manage sustained dual-role pressure. Even relatively small accommodations, especially during academically intensive periods such as rehearsals, performance examinations, auditions, or intensive practice periods, may help reduce the emotional burden associated with WSC and in turn reduce turnover risk (Naz et al., 2020).

This study has several limitations. First, its cross-sectional design limits causal inference. Although the model was theoretically grounded, the findings should be interpreted as associative rather than causal. Future longitudinal, multi-wave, or experience-sampling studies are needed to clarify the temporal ordering of these variables. Second, all variables were measured through self-report, which raises the possibility of social desirability and common method bias. Although multiple approaches were used to assess common method bias, the single-source design means that such bias cannot be entirely ruled out. Future research could strengthen the evidence by incorporating multi-source data. Third, although our focus on negative affect provides greater emotional specificity than broader constructs such as burnout, other mediating mechanisms may also be relevant, including frustration, guilt, or self-efficacy. Fourth, resilience was treated as a general personal resource and assessed using the brief two-item CD-RISC-2. Although this measure has shown acceptable psychometric properties and has been widely used in Chinese samples, its brevity may limit the breadth of construct coverage compared with longer resilience instruments. Future studies may benefit from using more comprehensive measures. Finally, the sample was limited to Chinese part-time music university students, and the study did not include a comparison group from other academic disciplines. This limits the extent to which we can determine whether the observed pattern is specific to music students or reflects student workers more generally. Future research should examine whether the observed pattern holds across different student groups and contexts.

## 5 Conclusions

The findings indicate that negative affect may represent one pathway linking WSC with turnover intention, suggesting that this association can be understood as a more proximal affective process rather than only a broad strain-based outcome. However, the findings did not support a moderated mediation pattern. Although resilience was hypothesized to moderate both the direct and indirect paths in this framework, only the direct association between WSC and turnover intention was significantly moderated, with the association being weaker at higher levels of resilience. These findings suggest that the association between WSC and students' intention to leave cannot be understood only in terms of competing role demands. It is also related to whether such conflict is associated with negative emotional experiences and to how personal resources are linked with students' responses to those experiences. More broadly, the study contributes by showing that the protective role of resilience may be more path-specific than uniformly generalized across the full conflict process. By focusing on music university students with part-time jobs, the study further extends WSC research to a structured, performance-based student-employment context in which academic demands are closely tied to professional development.

## Data Availability

The raw data supporting the conclusions of this article will be made available by the authors, without undue reservation.
