# Supplementary material for: Work–school conflict and turnover intention among Chinese Music University students with part-time jobs: the roles of negative affect and resilience
Source: Front Psychol. 2026 Jun 18;17:1849001. doi: 10.3389/fpsyg.2026.1849001 (PMC13323124; doi:10.3389/fpsyg.2026.1849001)
Supplement: Supplementary file 1 [file Table_1.docx]

Supplementary Table 1 Skewness and kurtosis values for continuous variables

| Variable | Skewness | kurtosis |
| --- | --- | --- |
| Age | 0.20 | -0.63 |
| Working hours per week | 0.10 | -0.79 |
| Work-school conflict | 0.32 | 0.56 |
| Negative affect | 0.14 | -0.27 |
| Resilience | -0.36 | 0.50 |
| Turnover intention | 0.43 | 0.61 |

Supplementary Table 2 Tolerance and variance inflation factor (VIF) values for predictors in the turnover intention model

| Variable | Tolerance | Variance Inflation Factor |
| --- | --- | --- |
| Work-school conflict | 0.46 | 2.18 |
| Negative affect | 0.61 | 1.65 |
| Resilience | 0.55 | 1.81 |
| Work-school conflict*Resilience | 0.65 | 1.53 |
| Negative affect*Resilience | 0.67 | 1.49 |
